# Supplementary material for: A Mousepad Triboelectric-Piezoelectric Hybrid Nanogenerator (TPHNG) for Self-Powered Computer User Behavior Monitoring Sensors and Biomechanical Energy Harvesting
Source: Polymers (Basel). 2023 May 26;15(11):2462. doi: 10.3390/polym15112462 (PMC10255169; doi:10.3390/polym15112462)
Supplement: Supplementary file 1 [file polymers-15-02462-s001.zip › supporting information.pdf]

polymers

Supporting Information

A Mousepad Triboelectric-Piezoelectric Hybrid Nanogenerator (TPHNG) for Self-Powered Computer User Behavior Monitoring Sensors and Biomechanical Energy Harvesting

Gang Jian [1,*], Ning Yang [2,*], Shangtao Zhu [2], Qingzhen Meng [2] and Chun Ouyang [2,3]

[1] Shenzhen Institute of Advanced Electronic Materials, Shenzhen Institutes of Advanced Technology, Chinese Academy of Sciences, Shenzhen 518055, China
[2] School of Materials Science and Engineering, Jiangsu University of Science and Technology, Zhenjiang 212100, China
[3] Wuxi Hansu Technology Co., Ltd., 216 Xitai Road, Wuxi 214111, China
* Correspondence: gjian@just.edu.cn (G.J.); ning15890317235@163.com (N.Y.)

Citation: Jian, G.; Yang, N.; Zhu, S.; Meng, Q.; Ouyang, C. A Mousepad Triboelectric-Piezoelectric Hybrid Nanogenerator (TPHNG) for Self-Powered Computer User Behavior Monitoring Sensors and Biomechanical Energy Harvesting. *Polymers* **2023**, *15*, 2462. https://doi.org/10.3390/polym15112462

Academic Editor: Amir Masoud Pourrahimi

Received: 6 April 2023
Revised: 17 May 2023
Accepted: 19 May 2023
Published: 26 May 2023

Copyright: © 2023 by the authors. Licensee MDPI, Basel, Switzerland. This article is an open access article distributed under the terms and conditions of the Creative Commons Attribution (CC BY) license (https://creativecommons.org/licenses/by/4.0/).

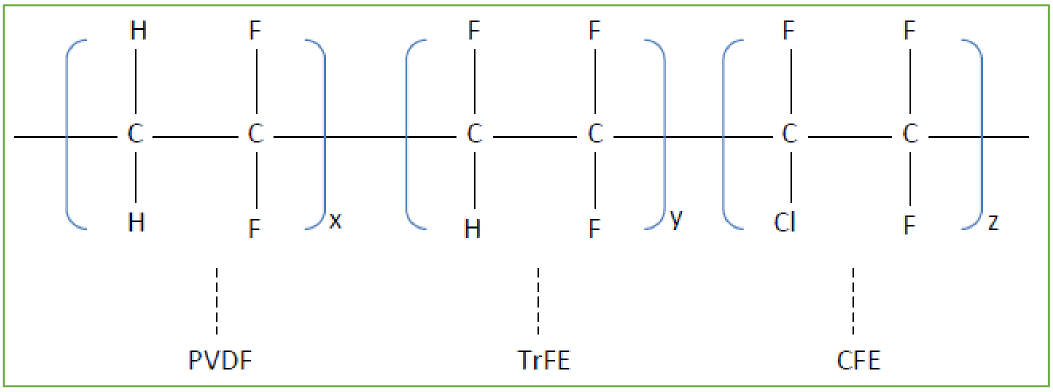

Figure S1. Molecule structure of the terpolymer P(VDF-TrFE-CFE).

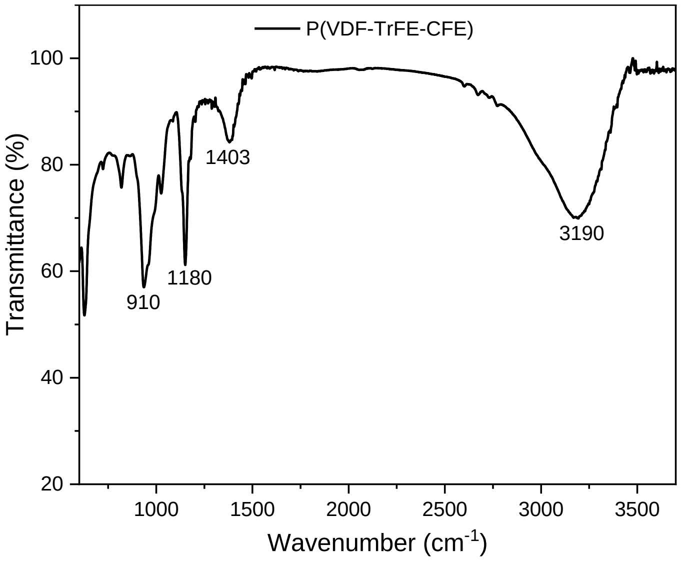

Figure S2. FTIR spectrum of the as-prepared P(VDF-TrFE-CFE) film, from which well-polymerized feature can be observed. C. Li, et al. Nanoscale Res. Lett. 15 (2020) 36.

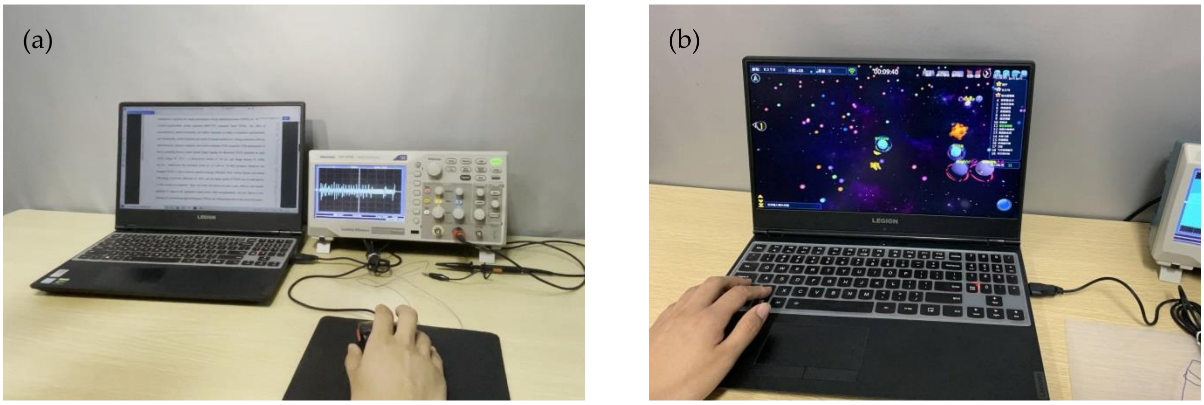

Figure S3. (a) Photograph of browsing a document. (b) Photograph of playing a computer game.

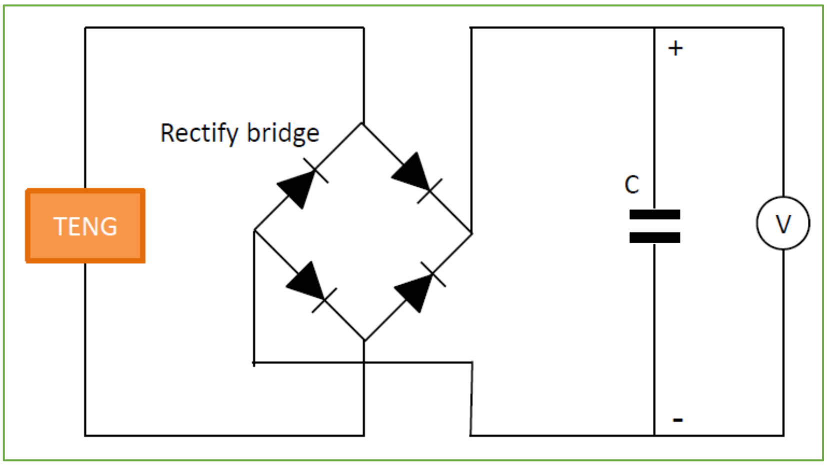

Figure S4. Schematic circuit of a full-wave bridge rectification for transforming AC electricity to DC.

Video S1. Voltage outputs of a hybrid nanogenerator for real time detecting human's motion of using a computer and the mouse.

Video S2. LEDs instantaneously driven by a hybrid nanogenerator upon sliding and clicking the mouse on the top of the device.
